# Analysis of Copper(II), Cobalt(II) and Iron(III) Sorption in Binary and Ternary Systems by Chitosan-Based Composite Sponges Obtained by Ice-Segregation Approach

**DOI:** 10.3390/gels7030103

**Published:** 2021-07-24

**Authors:** Maria Valentina Dinu, Doina Humelnicu, Maria Marinela Lazar

**Affiliations:** 1 “Mihai Dima” Department of Functional Polymers, “Petru Poni” Institute of Macromolecular Chemistry, Grigore Ghica Voda Alley 41A, 700487 Iasi, Romania; mariperju@icmpp.ro; 2 Faculty of Chemistry, “Al. I. Cuza” University of Iasi, Carol I Bd. 11, 700506 Iasi, Romania; doinah@uaic.ro

**Keywords:** heavy metal ion sorption, binary system, ternary system, copper(II), cobalt(II), iron(III), chitosan, composite sponges, recycling

## Abstract

With the intensive industrial activity worldwide, water pollution by heavy metal ions (HMIs) has become a serious issue that requires strict and careful monitoring, as they are extremely toxic and can cause serious hazards to the environment and human health. Thus, the effective and efficient removal of HMIs still remains a challenge that needs to be solved. In this context, copper(II), cobalt(II) and iron(III) sorption by chitosan (CS)-based composite sponges was systematically investigated in binary and ternary systems. The composites sponges, formed into beads, consisting of ethylenediaminetetraacetic acid (EDTA)- or diethylenetriaminepentaacetic acid (DTPA)-functionalized CS, entrapping a natural zeolite (Z), were prepared through an ice-segregation technique. The HMI sorption performance of these cryogenically structured composite materials was assessed through batch experiments. The HMI sorption capacities of CSZ-EDTA and CSZ-DTPA composite sponges were compared to those of unmodified sorbents. The Fe(III) ions were mainly taken up when they were in two-component mixtures with Co(II) ions at pH 4, whereas Cu(II) ions were preferred when they were in two-component mixtures with Co(II) ions at pH 6. The recycling studies indicated almost unchanged removal efficiency for all CS-based composite sorbents even after the fifth cycle of sorption/desorption, supporting their remarkable chemical stability and recommending them for the treatment of HMI-containing wastewaters.

## 1. Introduction

Heavy metal ions (HMIs) occur naturally in the earth’s crust. HMIs can enter into our bodies through food, drinking water and air. As essential elements, some HMIs (e.g., copper, zinc, iron) are vital in maintaining the metabolism of the human body when they are present in small amounts. At high concentrations, they are extremely toxic for living organisms or other biological systems [1]. The major sources of water pollution by HMIs are geological (natural) sources, mining and metal processing and industrial and domestic use of HMI salts (e.g., chromium in tanneries, copper and arsenic salts in pesticides or lead in petroleum products). Unfortunately, HMIs cannot be degraded and the only way to prevent their release into the environment is to recover and reuse them.

Copper ions ensure the proper function of many enzymes. However, copper salts are widely used as fungicides and germicides in agriculture and thus are commonly known as a source of risk for both humans and animals. Exceeding the tolerance level of 2.0 mg/L for copper can result in nausea, vomiting, diarrhea, liver poisoning and Wilson’s disease [2]. Cobalt is another essential metal for both humans and animals. It is an active nutrient for bacteria, algae and fungi. Nevertheless, high levels of cobalt ions in water and food can reduce thyroid function or cause low blood pressure, hyperglycemia, paralysis, and bone defects [3]. Iron plays also a key role in human nutrition, helping to form hemoglobin, which carries the oxygen to all cells. It is also involved in cell metabolism and can be found in many enzymes of the body. Iron deficiency leads to anemia, fatigue and infections. In contrast to copper and cobalt, the ingestion of iron from drinking water is not directly associated with negative health effects. However, the iron sediments may contain traces of impurities or bacteria that can induce certain health issues [4].

In this context, the discharge of HMIs from aqueous solutions has been intensively carried out using various processes, including chemical precipitation, extraction with different solvents, flocculation, ion exchange, ultrafiltration, reverse osmosis and adsorption [5,6,7,8,9,10]. Among these, adsorption is considered a simple and efficient technique to remove HMIs from wastewaters [8,9,10,11,12]. A large variety of sorbent materials, such as metal oxides/hydroxides [13,14], zeolites [15,16], commercial or synthetic activated carbon [17,18], biomass [19], synthetic organic supports [6,8,9], and unmodified or functionalized polysaccharides (chitosan, salecan, pullulan, alginate) [20,21,22,23,24,25,26,27,28,29] have been reported to remove HMIs from contaminated waters. 

Lately, the employment of inexpensive and eco-friendly sorbents, such as renewable natural polymers, in the extraction and recovery of certain HMIs has drawn considerable interest [2,3,10,11,20,21,22,23,24,25,26,27,28,29]. Amongst these low-cost sorbents, chitosan (CS) displayed promising abilities to bind different HMIs due to the presence of numerous amino and hydroxyl groups in its structure [2,20,27]. Moreover, to increase CS chelation performance for HMIs, the grafting of various functional groups (8-hydroxyquinoline [3], thiourea [21], carboxylic [23], histidine [24]), and the construction of ion-imprinted CS-based sorbents [25,30] have been reported. Nevertheless, most of these sorbents were tested for removal of HMIs from single-component aqueous systems. As wastewaters consist of more complex mixtures of HMIs, the necessity of extending the application range of eco-friendly sorbents based on renewable resources is eagerly demanded. To date, there are just a few studies focused on multicomponent aqueous mixtures [31,32,33]. In this regard, we used here composite sponges, formed into beads, based on ethylenediaminetetraacetic acid (EDTA)- or diethylenetriaminepentaacetic acid (DTPA)-functionalized chitosan (CS), entrapping a natural zeolite (Z), for removal of copper(II), cobalt(II) and iron(III) ions from binary and ternary aqueous mixtures. We systematically evaluated the influence of initial HMI concentration and pH on the sorption performance of CSZ-based sorbents. The HMI desorption and recycling abilities of CSZ-EDTA and CSZ-DTPA composites were also assessed in comparison to the unmodified CSZ sorbent.

## 2. Results and Discussion

**Figure 1:**
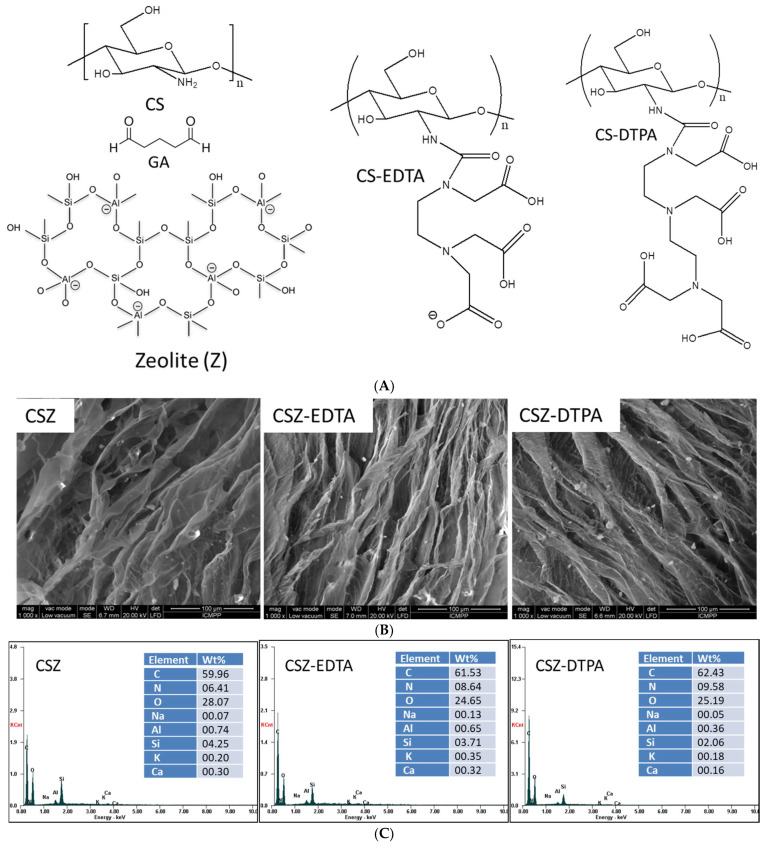
(**A**) Chemical structures of the chitosan (CS), glutaraldehyde (GA), zeolite (Z), ethylenediaminetetraacetic acid-functionalized CS (CS-EDTA) and diethylenetriaminepentaacetic acid-functionalized CS (CS-DTPA) used to prepare the composite materials tested as sorbents for copper(II), cobalt(II) and iron(III) removal from binary and ternary systems. (**B**) SEM micrographs of CSZ, CSZ-EDTA and CSZ-DTPA composite sorbents; (**C**) EDX profiles and the corresponding weight percentages of elements present on the surface of CSZ, CSZ-EDTA and CSZ-DTPA composite sorbents.

To ensure a fast removal of HMIs from contaminated waters, the features of sorbents and their abilities to strongly bind ionic species should be considered. It is known that chelating agents such as aminopolycarboxylic acids form stable structures with HMIs. In this context, our study aimed to use glutaraldehyde (GA) cross-linked CSZ composite sponges bearing EDTA or DTPA functional groups (Figure 1A) for removal of Cu(II), Co(II) and Fe(III) ions from two- or three-component aqueous mixtures. All CS-based composite sorbents obtained through the ice-segregation approach exhibited a porous structure with walls arranged along the freezing direction (Figure 1B), which could allow easy and rapid access of HMIs towards ligand moieties. Due to the particularities of cryogelation, where the cross-linking polymerization reaction occurs in a highly concentrated non-frozen micro-phase, elastic and sponge-like materials with shape memory features are generated. The elemental analysis on the surfaces of CSZ, CSZ-EDTA and CSZ-DTPA composite sponges was performed using an energy dispersive X-ray detector (EDX) (Figure 1C). The EDX profiles were consistent with the presence of C, N and O from CS and Na, Al, Si, K and Ca from natural zeolite. The Si/Al ratio within all CSZ-based composites was almost the same, indicating that the zeolite microparticles were not leached out during the functionalization process of CSZ matrix with EDTA or DTPA ligand groups (insets of Figure 1C). In addition, the weight percentage of N atoms increased for CSZ-EDTA and CSZ-DTPA composites, which proves the successful introduction of the EDTA and DTPA moieties.

**Figure 2:**
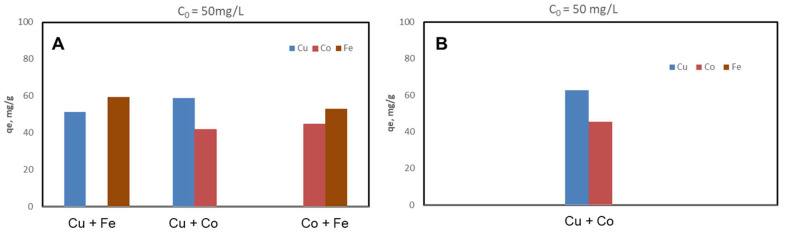
Sorption capacity and competitive sorption behavior of unmodified CS composite sorbent (CSZ) at pH = 4 (**A**) and pH = 6 (**B**) in a binary system. Sorbent dosage = 0.4 g L^−1^; contact time = 24 h; T = 21 ± 1 °C; initial Cu(II), Co(II) or Fe(III) concentration = 50 mg L^−1^. Note: At pH 6, only the system containing Cu and Co ions was studied because Fe ions precipitate at this value.

**Figure 3:**
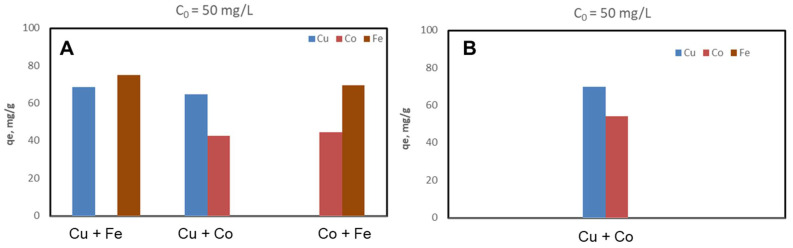
Sorption capacity and competitive sorption behavior of EDTA-functionalized CS composite sorbent (CSZ-EDTA) at pH = 4 (**A**) and pH = 6 (**B**) in a binary system. Sorbent dosage = 0.4 g L^−1^; contact time = 24 h; T = 21 ± 1 °C; initial Cu(II), Co(II) or Fe(III) concentration = 50 mg L^−1^. Note: At pH 6, only the system containing Cu and Co ions was studied because Fe ions precipitate at this value.

**Figure 4:**
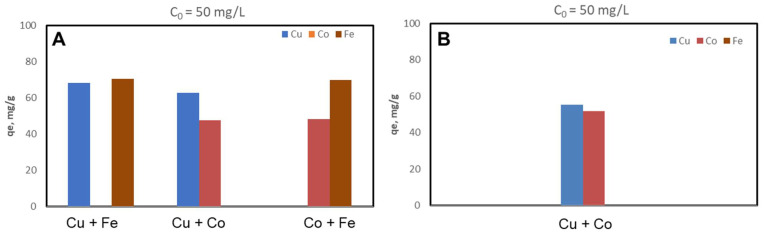
Sorption capacity and competitive sorption behavior of DTPA-functionalized CS composite sorbent (CSZ-DTPA) at pH = 4 (**A**) and pH = 6 (**B**) in a binary system. Sorbent dosage = 0.4 g L^−1^; contact time = 24 h; T = 21 ± 1 °C; initial Cu(II), Co(II) or Fe(III) concentration = 50 mg L^−1^. Note: At pH 6, only the system containing Cu and Co ions was studied because Fe ions precipitate at this value.

**Figure 5:**
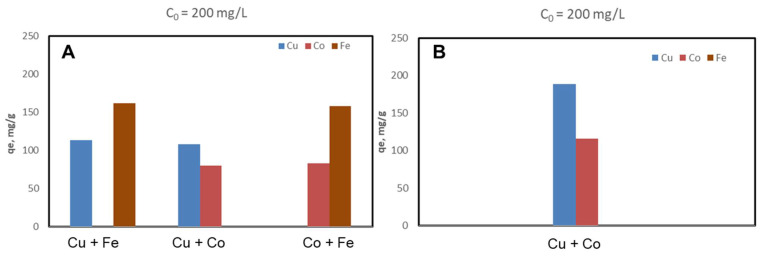
Sorption capacity and competitive sorption behavior of unmodified CS composite sorbent (CSZ) at pH = 4 (**A**) and pH = 6 (**B**) in a binary system. Sorbent dosage = 0.4 g L^−1^; contact time = 24 h; T = 21 ± 1 °C; initial Cu(II), Co(II) or Fe(III) concentration = 200 mg L^−1^. Note: At pH 6, only the system containing Cu and Co ions was studied because Fe ions precipitate at this value.

**Figure 6:**
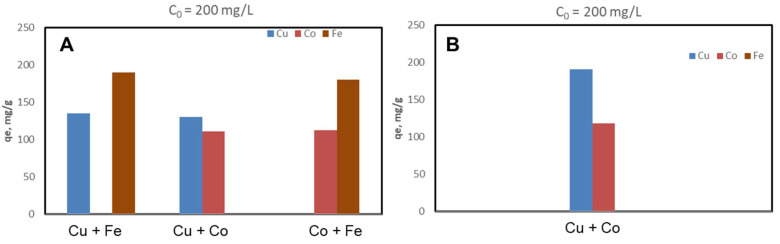
Sorption capacity and competitive sorption behavior of EDTA-functionalized CS composite sorbent (CSZ-EDTA) at pH = 4 (**A**) and pH = 6 (**B**) in a binary system. Sorbent dosage = 0.4 g L^−1^; contact time = 24 h; T = 21 ± 1 °C; initial Cu(II), Co(II) or Fe(III) concentration = 200 mg L^−1^. Note: At pH 6, only the system containing Cu and Co ions was studied because Fe ion precipitates at this value.

**Figure 7:**
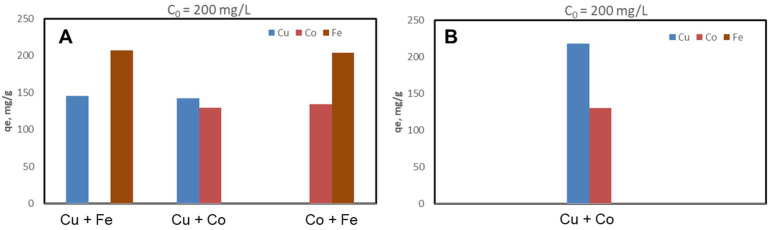
Sorption capacity and competitive sorption behavior of DTPA-functionalized CS composite sorbent (CSZ-DTPA) at pH = 4 (**A**) and pH = 6 (**B**) in a binary system. Sorbent dosage = 0.4 g L^−1^; contact time = 24 h; T = 21 ± 1 °C; initial Cu(II), Co(II) or Fe(III) concentration = 200 mg L^−1^. Note: At pH 6, only the system containing Cu and Co ions was studied because Fe ions precipitate at this value.

The CSZ, CSZ-EDTA and CSZ-DTPA composites were systematically tested for sorption of Cu(II), Co(II) and Fe(III) ions at initial HMIs concentration values of 50 mg/L (Figure 2, Figure 3 and Figure 4) and 200 mg/L (Figure 5, Figure 6 and Figure 7), respectively. The study was also performed at pH = 4 and pH = 6 using both binary and ternary systems.

### 2.1. Sorption of HMIs in a Binary System

The binding capacity of Cu(II), Co(II) and Fe(III) ions onto the CSZ-EDTA and CSZ-DTPA matrices was investigated in comparison to that of the unmodified CSZ sorbent. Batch experiments were first performed under competitive conditions using two-component synthetic aqueous mixtures. The sorption capacity and the competitive sorption behavior of all composites at pH = 4 and pH = 6 are presented in Figure 2, Figure 3 and Figure 4.

At pH = 4, the Fe(III) ions were generally preferred when they were in a two-component mixture with Co(II) ions (Figure 2A, Figure 3A and Figure 4A). However, when the Fe(III) ions were in a binary mixture with Cu(II) ions, all CS-based composites exhibited almost the same sorption capacity for both HMIs (Figure 2A, Figure 3A and Figure 4A). When the Cu(II) ions were in competition with Co(II) ions, the Cu(II) ions were preferentially adsorbed onto CSZ, CSZ-EDTA and CSZ-DTPA composite sorbents, irrespective of the aqueous solution pH (Figure 2B, Figure 3B and Figure 4B).

An increase of the sorption capacity was observed for the EDTA- and DTPA-modified CSZ sorbents. For instance, the EDTA-functionalized sorbent exhibited a sorption capacity of 68.77 mg Cu(II)/g and 75.07 mg Fe(III)/g, while the unmodified CSZ sorbent showed a sorption capacity of only 51.23 mg Cu(II)/g and 59.31 mg Fe(III)/g.

A similar behavior was also observed when the initial HMI concentration increased from 50–200 mg/L (Figure 5, Figure 6 and Figure 7).

All CS-based composite sorbents in acidic medium (pH = 4) showed a high affinity for Fe(III) ions when they were in mixture with Cu(II) or Co(II) ions (Figure 5A, Figure 6A and Figure 7A). The experimental q_e_ values for Fe(III) ion removal from their mixture with Cu(II) ions by CSZ, CSZ-EDTA and CSZ-DTPA sorbents were 161.60 mg/g, 189.61 mg/g and 206.65 mg/g, respectively. The q_e_ values for Fe(III) ions sorption slightly decreased when they were taken up from the binary system with Co(II). Thus, the amounts of Fe(III) retained by CS-based composite sorbents ranged from 157.56 mg/g to 203.75 mg/g (Figure 5A, Figure 6A and Figure 7A).

At pH = 6, Cu(II) ions were mainly retained over Co(II), and the Cu(II) sorption capacities increased from 188.31 mg/g for CSZ sorbent (Figure 5B) to 190.64 mg/g for CSZ-EDTA sorbent (Figure 6B) and to 218 mg/g for CSZ-DTPA sorbent (Figure 7B).

### 2.2. Sorption of HMIs in Ternary System

**Figure 8:**
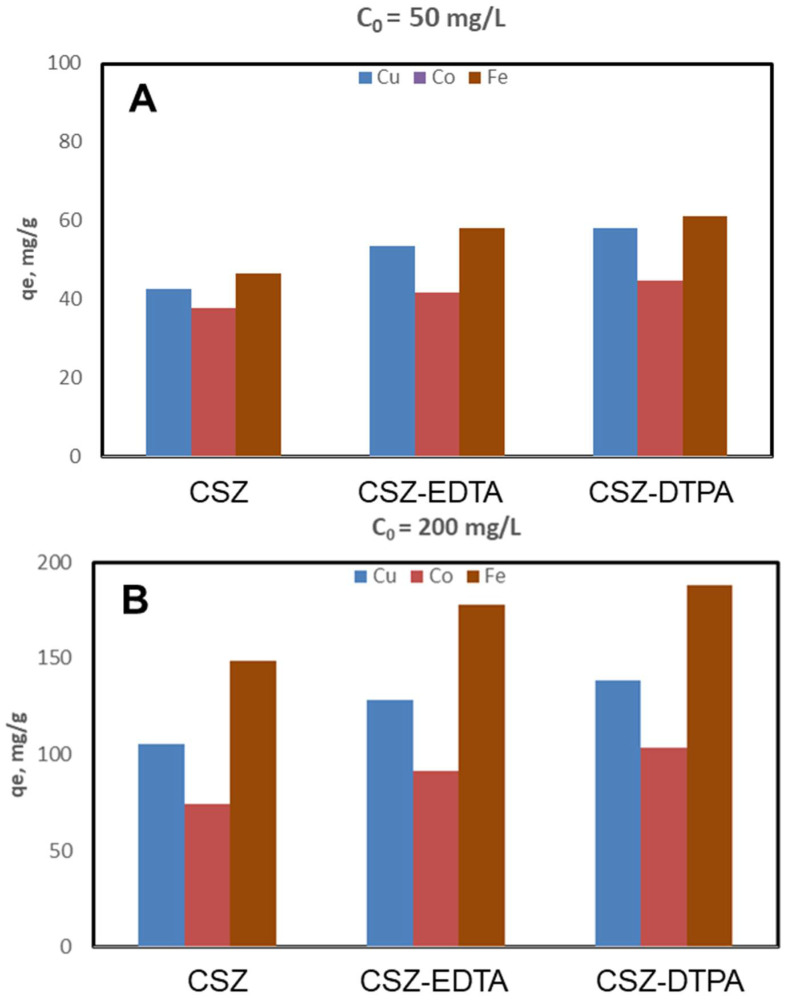
Sorption capacity and competitive sorption behavior of CSZ, CSZ-EDTA and CSZ-DTPA composite sorbents toward Cu(II), Co(II) and Fe(III) in a ternary system with an initial HMI concentration of 50 mg/L (**A**) or 200 mg/L (**B**). Sorbent dosage = 0.4 g L^−1^; pH = 4; contact time = 24 h; T = 21 ± 1 °C.

The study for the ternary system was only performed at pH = 4, using an initial HMI concentration of 50 mg/L (Figure 8A) and of 200 mg/L (Figure 8B). At pH = 6 it was not possible to prepare a ternary system since Fe(III) ions precipitate at this value.

**Figure 9:**
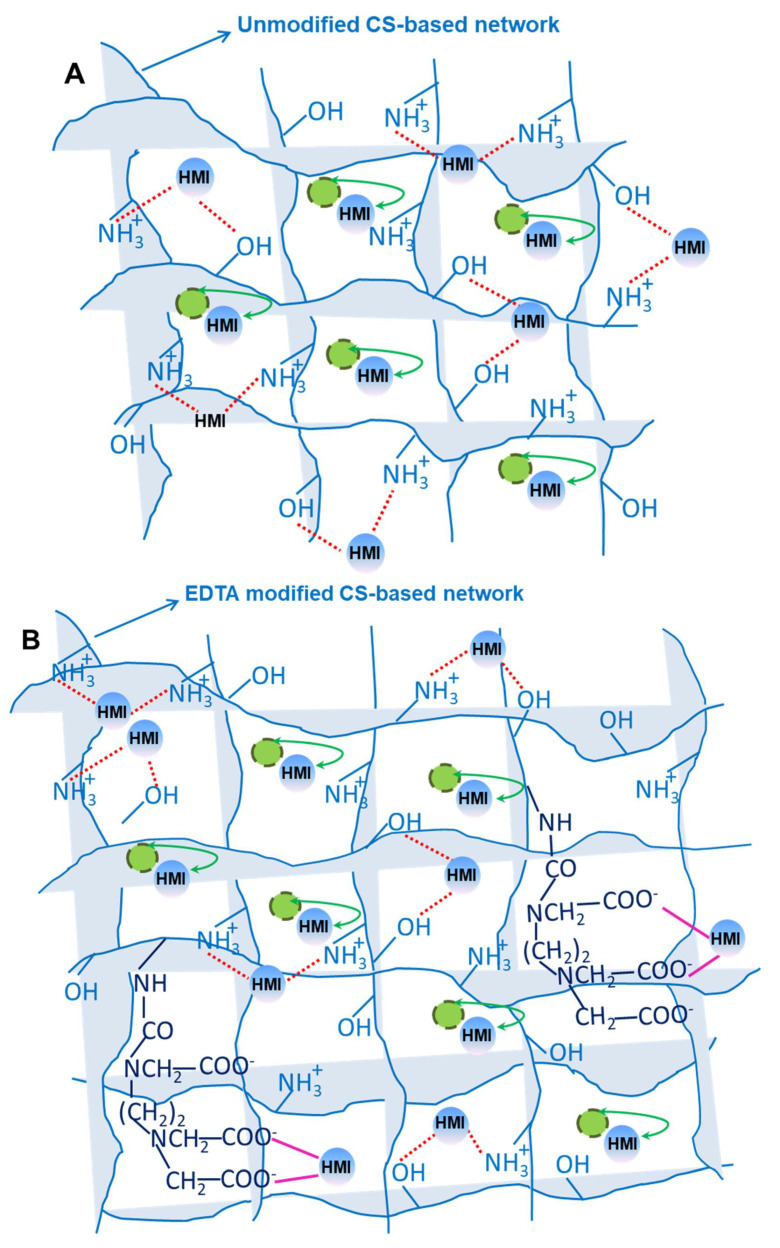

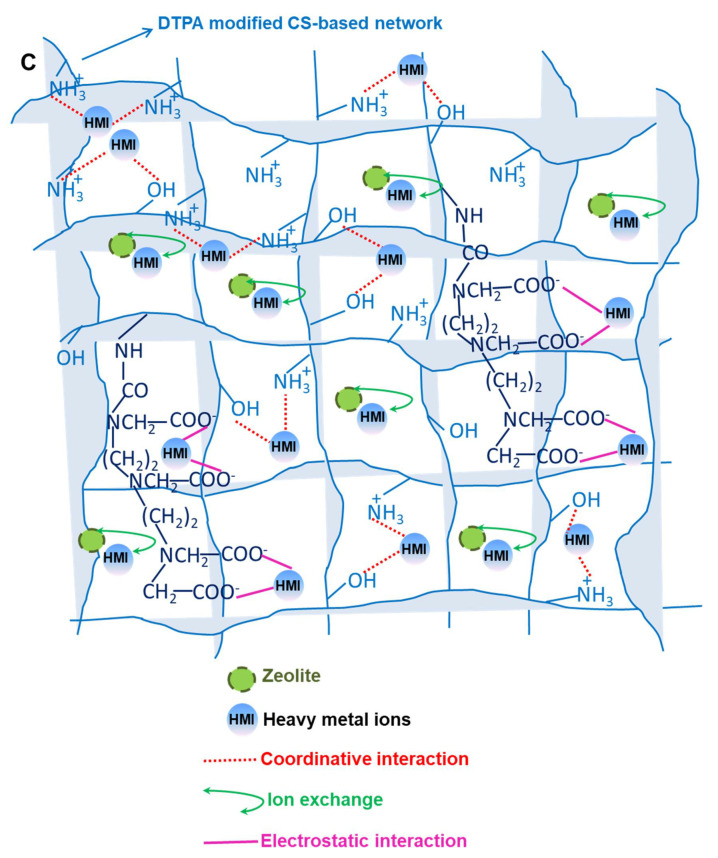
A schematic representation of the possible interactions between HMIs and the functional groups of each composite sponge: (**A**) CSZ sorbent; (**B**) CSZ-EDTA sorbent; and (**C**) CSZ-DTPA sorbent.

Comparing the q_e_ values of CSZ, CSZ-EDTA and CSZ-DTPA composite sorbents, the CSZ-DTPA showed the highest sorption capacity for Fe(III) ions, i.e., 61.1 mg/g (Figure 8A) and 188.03 mg/g (Figure 8B). The affinity sequence of all CS-based composites was: Fe(III) > Cu(II) > Co(II), regardless of the pH of the aqueous mixture. The possible interactions between HMIs and the functional groups of composite sponges are depicted in Figure 9. The most active coordination centers for HMIs are the -NH_2_ and -OH groups of CS (Figure 9A). The –COOH groups from EDTA or DTPA bind the HMIs throughout the electrostatic interactions (Figure 9B,C), while the natural zeolite is involved in an ion exchange process for HMI binding.

### 2.3. HMIs Desorption and Sorbent Recycling

The recycling ability of the sorbents and the recovery of HMIs are very important factors that should be assessed in order to evaluate the cost-effectiveness of a sorption process in wastewater treatment [31,33]. In this study, the HMIs adsorbed onto CS-based composite sorbents were eluted with 0.1 M HCl solution and, before another sorption cycle, the sorbents were regenerated with 0.1 M NaOH. 

**Figure 10:**
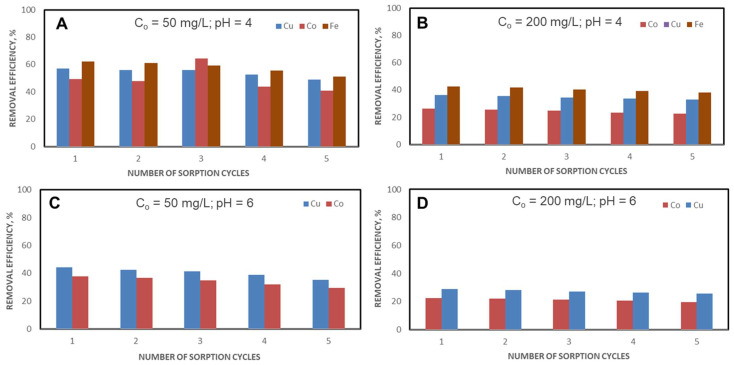
Influence of successive sorption/desorption cycles on HMIs removal from ternary (**A**,**B**) or binary mixtures (**C**,**D**) using unmodified CSZ sorbent. (**A**) C_o_ = 50 mg/L; pH = 4; (**B**) C_o_ = 200 mg/L; pH = 4; (**C**) C_o_ = 50 mg/L; pH = 6; (**D**) C_o_ = 200 mg/L; pH = 6.

**Figure 11:**
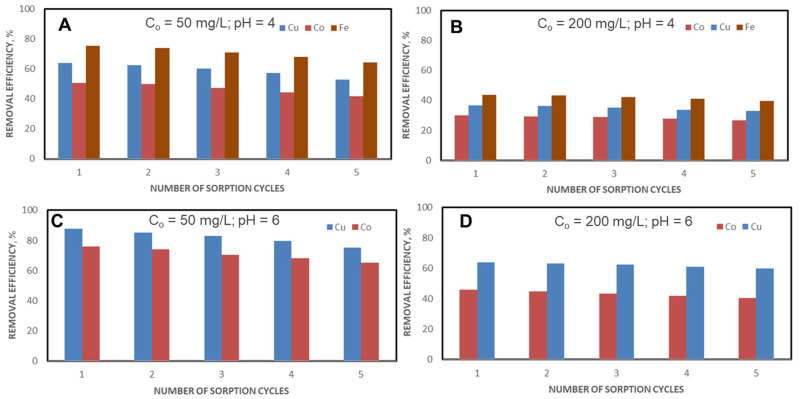
Influence of successive sorption/desorption cycles on HMIs removal from ternary (**A**,**B**) or binary mixtures (**C**,**D**) using EDTA-functionalized CSZ sorbent. (**A**) C_o_ = 50 mg/L; pH = 4; (**B**) C_o_ = 200 mg/L; pH = 4; (**C**) C_o_ = 50 mg/L; pH = 6; (**D**) C_o_ = 200 mg/L; pH = 6.

**Figure 12:**
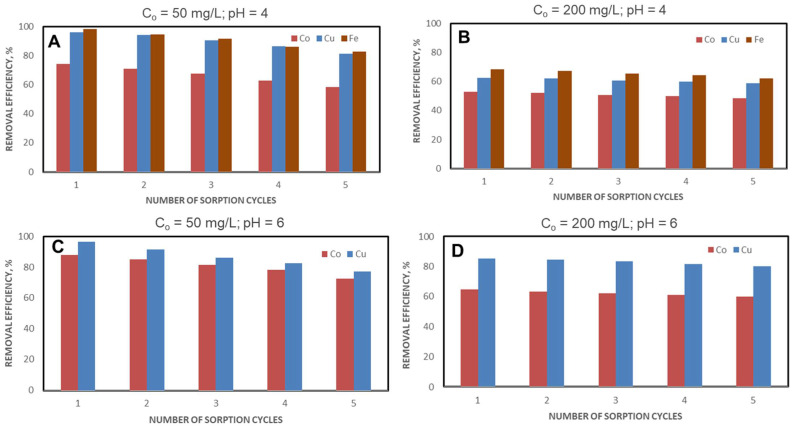
Influence of successive sorption/desorption cycles on HMIs removal from ternary (**A**,**B**) or binary mixtures (**C**,**D**) using DTPA-functionalized CSZ sorbent. (**A**) C_o_ = 50 mg/L; pH = 4; (**B**) C_o_ = 200 mg/L; pH = 4; (**C**) C_o_ = 50 mg/L; pH = 6; (**D**) C_o_ = 200 mg/L; pH = 6.

The CSZ, CSZ-EDTA and CSZ-DTPA composite sorbents were involved in five successive sorption/desorption cycles using either pH 4 (Figure 10A,B, Figure 11A,B and Figure 12A,B) or pH 6 (Figure 10C,D, Figure 11C,D and Figure 12C,D) and an initial HMI concentration of 50 mg/L (Figure 10A,C and Figure 11A,C) or 200 mg/L (Figure 10B,D, Figure 11B,D and Figure 12B,D).

Table 1 summarizes the experimental data obtained on HMIs removal from ternary (A,B) and binary mixtures (C,D) using unmodified CSZ sorbent, EDTA-functionalized CSZ sorbent and DTPA-functionalized CSZ sorbent after the first and the fifth cycles of sorption.

Analyzing the removal efficiency (*RE*) values, it should be pointed out that better results were obtained when an initial HMI concentration of 50 mg/L and EDTA- or DTPA-functionalized sorbents were used (Figure 10, Figure 11 and Figure 12 and Table 1). Thus, at pH = 4, in the case of the unmodified CSZ sorbent, the percentage of Cu(II) ions retained decreased by 17.69% at the initial HMI concentration of 50 mg/L (Figure 10A) and by 9.41% for an initial HMI concentration of 200 mg/L (Figure 10B). However, for the unmodified CSZ sorbent, after the first cycle of sorption, removal efficiencies of only 57.32%, 49.38% and 62.16% were achieved for Cu(II), Co(II) and Fe(III) ions at an initial HMI concentration of 50 mg/L and pH of 4 (Figure 10A and Table 1). When the concentration increased to 200 mg/L, the removal efficiency values for this sorbent drastically decreased to about 36.33% for Cu(II) ions, 26.48% for Co(II) ions and 42.63% for Fe(III) ions (Figure 10B and Table 1). A lower removal efficiency was also observed for the control sorbent (unmodified CSZ sample) even at pH 6, irrespective of the initial HMI concentration (Figure 10C,D, and Table 1). By contrast, as Figure 11 and Figure 12 and Table 1 show, the removal efficiency values of HMIs with EDTA- or DTPA-functionalized composite sorbents significantly increased compared to unmodified CSZ sorbents. In the ternary system, at an initial HMI concentration of 50 mg/L and pH 4, the removal efficiency was about 64.14% for Cu(II) ions, 50.88% for Co(II) ions and 75.32% Fe(III) ions when CSZ-EDTA sorbents were used (Figure 11A and Table 1). In the case of CSZ-DTPA sorbents, almost complete removal was observed for Cu(II) ions (96.04%) and Fe(III) ions (98.26%) (Figure 12A and Table 1). By raising the initial HMI concentration to 200 mg/L (Figure 11B and Figure 12B and Table 1), the values of the removal efficiency of the Cu(II), Co(II) and Fe(III) ions were slightly diminished, as expected. In the binary system, at an initial HMI concentration of 50 mg/L and pH 6, the removal efficiency with CSZ-EDTA sorbent was about 87.54% for Cu(II) ions and 75.94% for Co(II) ions (Figure 11C and Table 1), while with CSZ-DTPA sorbent the removal efficiency was around 96.84% for Cu(II) ions and 88.12% for Co(II) ions (Figure 12C and Table 1). At a higher initial HMI concentration (200 mg/L) and pH 6, Cu(II) ions were preferentially desorbed with removal efficiencies of 63.68% and 85.27% by CSZ-EDTA sorbent (Figure 11D and Table 1) and CSZ-DTPA sorbent, respectively (Figure 12D and Table 1).

Nevertheless, as can be seen from Figure 10, Figure 11 and Figure 12 and Table 1, there was a high regeneration capacity, i.e., the removal efficiency values remained almost unchanged for all CS-based composite sorbents even after the fifth cycle of sorption/desorption, indicating their remarkable chemical stability under HMI leaching and sorbent regeneration conditions.

The values of the sorption data obtained in this study for CSZ, CSZ-EDTA and CSZ-DTPA sponges were compared with those reported for other sorbents in Table 2.

Although all the sorbents included in Table 2 can be exploited for the removal of HMIs from multicomponent aqueous mixtures, it is important to point out that the choice of the best sorbent material should rely on various aspects, including the composition of the aqueous mixtures, the pH of the solution, the initial concentration of the HMIs and the sorbent dose. The values of qe for CSZ, CSZ-EDTA and CSZ-DTPA composite sorbents were comparable and even higher than those previously reported for other sorbents, supporting their great potential for application in the treatment of wastewaters containing HMIs.

## 3. Conclusions

The sorption performances of ethylenediaminetetraacetic acid (EDTA)- and diethylenetriaminepentaacetic acid (DTPA)-functionalized CS composite sponges, entrapping a natural zeolite, towards the removal of Cu(II), Co(II) and Fe(III) ions from binary or ternary aqueous mixtures was evaluated in depth in this work in comparison to those of unmodified CS–based sorbents. The effect of the initial HMI concentration and pH on the sorption features is well-established. All CS-based composite sorbents in acidic medium (pH = 4) exhibited a high affinity for Fe(III) ions when they were in a mixture with Cu(II) or Co(II) ions. The experimental q_e_ values for removal of Fe(III) ions from their mixture with Cu(II) ions by CSZ, CSZ-EDTA and CSZ-DTPA sorbents were 161.60 mg/g, 189.61 mg/g and 206.65 mg/g, respectively. The q_e_ values for Fe(III) ion sorption slightly decreased when they were adsorbed from the binary system with Co(II). Thus, the amounts of Fe(III) retained by CS-based composite sorbents ranged from 157.56 mg/g to 203.75 mg/g. The HMI desorption and sorbent recycling studies demonstrated that better results are obtained when an initial HMI concentration of 50 mg/L and EDTA- or DTPA-functionalized sorbents are used. In the ternary system, at an initial HMI concentration of 50 mg/L and pH 4, the removal efficiency was about 64.14% for Cu(II) ions, 50.88% for Co(II) ions and 75.32% for Fe(III) ions when CSZ-EDTA sorbents were used. In the case of CSZ-DTPA sorbents, an almost complete removal was observed for Cu(II) ions (96.04%) and Fe(III) ions (98.26%). Moreover, a high regeneration capacity was observed for all CS-based composite sorbents even after the fifth cycle of sorption/desorption, which clearly indicates their remarkable chemical stability and potential application in wastewater treatment. As real-life aqueous effluents are composed of more complex mixtures of contaminants than the investigated systems, ongoing experiments will be dedicated to evaluate their removal by the newly prepared composite sponge sorbents. In addition, the beads can be easily prepared and the next step is their testing under dynamic operation conditions. 

## 4. Materials and Methods

### 4.1. Materials

Chitosan (CS) with a relative viscosity-average molecular weight of 342 kDa and a deacetylation degree (DD) of 85% was purchased from Sigma-Aldrich. Glutaraldehyde (GA) solution at a concentration of 25% (w/w) in H_2_O was used as cross-linker and was also acquired from Sigma-Aldrich. The zeolite fraction, of sizes in the 0.032–0.050 mm range, was obtained from the volcanic tuffs cropping out in the Macicas area (Cluj County, Romania) and was used as inorganic filler within the CS matrix. 4,4′–Ethylenebis(2,6—morpholinedione) (EDTA dianhydride (EDTAD)) and N,N-Bis [2—(2,6-dioxomorpholino)ethyl]glycine (DTPA dianhydride (DTPAD)), provided by Sigma-Aldrich, were used as received, without any further purification.

CoCl_2_∙6H_2_O, CuSO_4_∙5H_2_O and Fe(NO_3_)_3_∙9H_2_O were used as sources of HMIs and were purchased from Sigma-Aldrich. Hydrochloric acid, sodium hydroxide, acetic acid and methanol of the highest commercial purity, provided by Chemical Company, Romania, were used as received.

### 4.2. Methods

#### 4.2.1. Preparation and Functionalization of CSZ Composite Sponges

Chemically cross-linked CS-based sorbents, formed into beads, were prepared in the presence of a natural zeolite using the ice-segregation technique, which consisted of a cryogenic process with three stages: freezing in liquid nitrogen, storage in the frozen state for a certain time, and defrosting, following a procedure previously reported [32,39] with some modifications. Thus, 2 g of CS (324 kDa, 85% DD) was dissolved in 100 mL of 1 wt.% acetic acid aqueous solution. Afterwards, 0.75 g of natural zeolite was added under vigorous stirring. Finally, 3.2 mL of GA (5 wt.%) was added dropwise over 30 min. The homogeneous dispersion obtained after 1 h of vigorous stirring was added drop-by-drop, with an Eppendorf pipette, into liquid nitrogen (LN). The frozen droplets were separated from the LN and immediately transferred to an Arctiko Freezer at −18 °C to ensure the complete cross-linking of CS by GA. After 24 h, the CS beads were thawed at room temperature for 1 h and washed several times with MilliQ water. The CSZ beads were freeze-dried in a LABCONCO FreeZone apparatus for 48 h, at –50 °C and 0.04 mbar. Ethylenediaminetetraacetic acid (EDTA) or diethylenetriaminepentaacetic acid (DTPA) ligand moieties were generated onto the CSZ composite sponges by reaction with EDTAD or DTPAD in a 1:1 *v*/*v* acetic acid–methanol mixture, following a procedure previously reported [40].

#### 4.2.2. Morphology and Elemental Surface Composition

The cross-sectional microstructure of the composite sponges was observed with a Quanta 200-FEI-type environmental scanning electron microscope (ESEM) at 20 kV in low vacuum mode. An energy dispersive X-ray (EDX) detector was used to map the elements and to estimate their ratio and distribution within the CSZ-based composites.

#### 4.2.3. Sorption Studies

The sorption performance of the CSZ-based composite sorbents was examined under competitive conditions, i.e., using a two- or three-component mixture of Cu(II), Co(II) and Fe(III) ions in batch mode. The effect of the initial HMI concentration (50 mg/L and 200 mg/L), as well as the influence of the solution pH (4 or 6), was investigated, while the other parameters were kept constant. The solutions were equilibrated for 24 h at 200 rpm. The final HMI concentrations were determined by flame atomic absorption spectrometry (FAAS) using a high-resolution ContrAA 300 Analytik Jena spectrometer equipped with a xenon lamp as a continuum radiation source. During experiment an aspiration rate of about 5 mL min^−1^ was used. The residual concentration of each HMIs was determined at the characteristic maximum wavelengths of 240 nm for Co(II) ions, 324 nm for Cu(II) ions and 248 nm for Fe(III) ions, respectively. The amount of HMI adsorbed at equilibrium (q_e_, mg g^−1^) on all CSZ-based sorbents was calculated as:

(1) $$q_{e}=\frac{\left(C_{0}-C_{e}\right)\times V}{m}$$

where *C_o_*—initial HMI concentration, mg L^-1^; *C_e_*—concentration of the HMIs in aqueous solution at equilibrium, mg L^−1^; *V*—volume of aqueous solution, L; and *m*—sorbent dose, g.

#### 4.2.4. Desorption and Reusability Experiments

In order to investigate the sorbent reusability, the HMIs loaded onto CSZ-based sorbents were eluted with 0.1M HCl aqueous solution. Then, the CSZ-based sorbents were washed several times with distilled water and were regenerated with 0.1M NaOH aqueous solution. After this treatment, the sorbents were reused in another cycle of sorption. The residual concentration of each HMI was determined by FAAS.

The efficiency of HMI removal (RE, %) from aqueous solution on all CSZ-based sorbents was calculated as:

(2) $$RE\left(\%\right)=\frac{C_{0}-C_{e}}{C_{0}}\times 100$$

where *C_o_* and *C_e_* have the same meanings as in Equation (1).

## Figures and Tables

**Table 1:** The removal efficiencies (*RE, %*) of the composite sponges after the first and the fifth cycles of sorption.

**Sorbents/pH**	**Removal Efficiency (*RE, %*) after the First Cycle of Sorption**					
	**C_0_ = 50 mg/L**			**C_0_ = 200 mg/L**		
	**Cu**	**Co**	**Fe**	**Cu**	**Co**	**Fe**
CSZ/pH 4	57.32	49.38	62.16	36.33	26.48	42.63
CSZ-EDTA/pH 4	64.14	50.88	75.32	36.79	30.08	43.83
CSZ-DTPA/pH 4	96.04	74.18	98.26	62.69	52.82	68.53
CSZ/pH 6	44.08	37.77	-	28.71	22.44	-
CSZ-EDTA/pH 6	87.54	75.94	-	63.68	45.72	-
CSZ-DTPA/pH 6	96.84	88.12	-	85.27	64.58	-
**Sorbents/pH**	**Removal Efficiency (*RE, %*) after the Fifth Cycle of Sorption**					
	**C_0_ = 50 mg/L**			**C_0_ = 200 mg/L**		
	**Cu**	**Co**	**Fe**	**Cu**	**Co**	**Fe**
CSZ/pH 4	49.15	41.02	51.26	32.91	22.64	38.22
CSZ-EDTA/pH 4	52.86	41.92	64.34	32.87	26.72	39.72
CSZ-DTPA/pH 4	81.26	58.36	82.72	58.82	48.66	62.26
CSZ/pH 6	35.04	29.42	-	25.62	19.42	-
CSZ-EDTA/pH 6	75.26	65.24	-	59.83	40.31	-
CSZ-DTPA/pH 6	77.44	72.66	-	80.13	59.83	-

**Table 2:** Comparative sorption data for removal of HMIs from multicomponent aqueous mixtures by various sorbents.

Sorbents	Type of Multicomponent Mixture	HMI	pH	C_0_, mg/L	Sorbent Dose, g/L	q_e_, mg/g	Refs.
Granular activated carbon (GAC)	Ternary	Al(III) Fe(III) Mn(II)	5	26–122	2	106.5 14.8 7.6	[34]
Amberlite IR-120H	Ternary	Al(III) Fe(III) Mn(II)	5	26–122	2	108.7 15.6 8.7	[34]
Activate carbon	Ternary	Cu(II) Ni(II) Zn(II)	5.5	10–100	2.5	18.6 16.1 12.1	[35]
CS-coated perlite beads	Binary	Cu(II) Ni(II)	5	50–200	2.5	147.0 38.9	[36]
	Binary	Cu(II) Co(II)				156.2 39.8	
	Binary	Ni(II) Co(II)				56.1 66.6	
	Ternary	Cu(II) Co(II) Ni(II)				128.2 35.2 30.4	
Cu^2+^-ion-imprinted polymer	Binary	Cu(II) Ni(II)	6	30–90	3	53.1 7.7	[37]
	Binary	Cu(II) Zn(II)				40.2 3.3	
	Binary	Cu(II) Pb(II)				38.4 128.1	
SBA-15 mesoporous silica	Quaternary	Cu(II) Ni(II) Co(II) Zn(II)	4.8		1	8.2 7.6 4.7 5.5	[38]
N-propylsalicylaldimino-functionalized SBA-15 mesoporous silica	Quaternary	Cu(II) Ni(II) Co(II) Zn(II)	4.8		1	44 3.5 2.9 2.6	[38]
Acid-activated Romanian zeolite	Five-component	Cu(II) Fe(III) Ni(II) Zn(II) Cr(III)	5 4 4.5 5 3.5	50–1000	3.5	9.7 7.3 2.2 2.2 0.12	[11]
CS/acid-activated Romanian zeolite composite cryogels	Five-component	Cu(II) Fe(III) Ni(II) Zn(II) Cr(III)	5 4 4.5 5 3.5	50–1000	3.5	61.1 18.6 12.2 53.4 0.8	[11]
CSZ	Binary	Cu(II) Fe(III)	4	200	0.4	113.3 161.6	This study
	Binary	Cu(II) Co(II)	4	200	0.4	107.8 80.3	
	Binary	Co(II) Fe(III)	4	200	0.4	82.8 157.5	
	Binary	Cu(II) Co(II)	6	200	0.4	188.3 115.3	
CSZ-EDTA	Binary	Cu(II) Fe(III)	4	200	0.4	135.3 189.6	
	Binary	Cu(II) Co(II)	4	200	0.4	130.7 110.6	
	Binary	Co(II) Fe(III)	4	200	0.4	112.2 180.2	
	Binary	Cu(II) Co(II)	6	200	0.4	190.64 118.0	
CSZ-DTPA	Binary	Cu(II) Fe(III)	4	200	0.4	145.4 206.7	
	Binary	Cu(II) Co(II)	4	200	0.4	141.9 129.1	
	Binary	Co(II) Fe(III)	4	200	0.4	133.8 203.7	
	Binary	Cu(II) Co(II)	6	200	0.4	218.0 130.4	
CSZ	Ternary	Cu(II) Co(II) Fe(III)	4	200	0.4	105.7 74.5 148.6	
CSZ-EDTA	Ternary	Cu(II) Co(II) Fe(III)	4	200	0.4	128.2 91.5 177.7	
CSZ-DTPA	Ternary	Cu(II) Co(II) Fe(III)	4	200	0.4	138.6 103.5 188.0	

## Data Availability

Not applicable.
